# Distal insertion rupture of lateral ankle ligament as a predictor of weakened and delayed sports recovery after acute ligament repair: mid-term outcomes of 117 cases

**DOI:** 10.1186/s12891-022-05260-6

**Published:** 2022-03-28

**Authors:** Mingze Du, Jun Li, Chen Jiao, Qinwei Guo, Yuelin Hu, Dong Jiang

**Affiliations:** 1 Department of Sports Medicine, Peking University Third Hospital, Institute of Sports Medicine of Peking University, Beijing Key Laboratory of Sports Injuries, Beijing, 100191 China; 2 Department of Joint Sports Medicine, Tai’an Central Hospital of Shandong Province, Tai’an, Shandong, 271099 China

**Keywords:** Acute ankle sprain, Lateral ankle ligament, Anatomic ligament repair, Return to sports, Arthroscopy

## Abstract

**Background:**

For lateral ankle ligament rupture, surgical repair has been considered for patients requiring return to high-demanding sports. However, there is a lack of systematic research regarding arthroscopic treatment followed by ligament repair for severe acute ankle sprain. The purpose of this study was to analyze the mid-term outcomes of arthroscopy followed by open anatomic lateral ankle ligament repair surgery for acute lateral ankle sprain and the impact of ligament rupture site on the outcomes.

**Methods:**

Professional or amateur athletes with clinically- and radiologically-confirmed grade III acute lateral ankle ligament injuries undergoing ankle arthroscopy followed by open anatomic ligament repair between June 2007 and May 2017 were reviewed. Intra-articular lesions and the location of rupture were first examined under arthroscopy. Simple suture repair was performed for mid- substance ligament rupture (middle group), while suture repair with anchors were used for the ruptures near the ligament attachment site on the fibular (proximal group), talar or the calcaneal side (distal group). Outcomes were evaluated at final follow-up, including visual analog scale (VAS) pain score, American Orthopaedic Foot & Ankle Society (AOFAS) score, Tegner score, time to return to sports, resumption of pre-injury sports level, sprain recurrence and range of motion (ROM).

**Results:**

A total of 117 patients were included for analysis, with a mean follow-up duration of 46.4 ± 16.1 months. There were 48 (41%) cases in the proximal group, 41 (35%) cases in the middle group and 28 (24%) cases in the distal group respectively. At final follow-up, all of the VAS score, AOFAS score and the Tegner score were significantly improved from the pre-operative level (*p* < 0.001). 12 (10%) patients complained of sprain recurrence during follow-up. 14 (12%) patients reported mild ROM restriction and 7 (6%) patients experienced transient skin numbness. The average time to return to pre-injury sports was 4.34 ± 1.11 months. The smallest proportion (86% ± 13%) of resumption of pre-injury sports level was reported from the distal group, compared with 93% ± 12% for the proximal group and 89% ± 14% for the middle group (*p* = 0.037). Time to return to sports was significantly longer for the distal group, with an average of 4.59 ± 1.27 months compared to 3.99 ± 1.09 months for the proximal group and 4.58 ± 0.90 months for the middle group (*p* = 0.009).

**Conclusions:**

Ankle arthroscopy followed by open anatomic ligament repair is a reliable procedure for patients requiring return to high demanding sports after severe acute ankle sprains. Distal rupture near the talar or calcaneal end was associated with delayed return-to-sport and inferior performance in resuming pre-injury sports level.

## Introduction

Ankle sprain is one of the most common sports injuries [1], accounting for 7–10% of patients presenting to the emergency department [2]. Improper management of ankle sprains can increase the risk of developing chronic ankle instability [3]. During the last decade, rehabilitation with optimal loading in brace was advocated for most ankle sprains [4]. However, treatment for grade III injuries is controversial. Surgical repair has been considered for patients requiring return to high demanding sports [5] to achieve stronger ankle stability and allow for an earlier return to sports.

Previous studies reported a high rate (10%–30%) of complications after open surgical repair of acute lateral ankle ligament rupture, including range of motion (ROM) restriction, wound problems, and nerve injuries, etc [6]. However, most of these reports were studies performed before the advent of arthroscopy. Reports on the mid- to long-term outcomes were also limited [7]. Recently, ankle arthroscopy has been recommended for better evaluation and management of associated intra-articular injuries [8, 9]. In addition, for acute ankle sprains, the location of ligament rupture can be observed under arthroscopy, facilitating optimal choice of incision and thus reducing the length of the incision and associated complications [10]. However, there is a lack of research on the mid- to long-term outcomes regarding arthroscopic treatment followed by ligament repair for severe acute ankle sprain.

The anterior talofibular ligament (ATFL) and the calcaneofibular ligament (CFL) are the most commonly involved ligaments in ankle sprains [11]. Previous studies indicated that due to differences in anatomy, there are differences in the healing time and healing ability of different injured parts which may affect the postoperative rehabilitation and outcomes, especially the time and degree of return to sports [12]**.** However, so far there is no research on the impact of the rupture site on the mid-term outcomes of the operation.

In the present study, patients with grade III acute lateral ankle ligament injuries underwent arthroscopy followed by open anatomic ligament repair. The purpose of this study was to evaluate the mid-term outcomes of this procedure and the impact of ligament rupture site on the outcomes. It was hypothesized that concurrent arthroscopy and open ligament repair would achieve overall good mid-term results and the outcomes might differ at different rupture sites. The study could also provide a reference for postoperative rehabilitation after acute lateral ankle ligament repair.

## Materials and methods

### Patients recruitment

Between June 2007 and May 2017, professional or amateur athletes with grade III acute lateral ankle ligament injuries were given the option of surgical repair. After thoroughly informed of the associated risks and predicted outcomes of both surgical and conservative treatments, patients opting for surgical management underwent arthroscopy and open anatomic ligament repair. Lateral ankle ligament injuries were confirmed by clinical examinations and radiological assessments. Depending upon the severity of the injury and the symptoms present, ligament injuries were graded as follows: Grade I: ligaments were strained but not ruptured, and the ankle was relatively stable; Grade II: partial ligament rupture, with varying degrees of ankle instability; Grade III: one or more ligaments were completely ruptured, which may cause fractures of surrounding bone structures and ankle instability [13–15].

Patients were included if the interval from trauma to surgery was less than 2 weeks. Patients were excluded if they had: (1) fracture requiring internal fixation; (2) osteochondral lesions (OCLs) requiring tissue transplantation rather than debridement or microfracture; (3) history of previous operations to the index ankle; or (4) history of sprain on the contralateral ankle. The research was approved by the IRB Medical Committee (IRB00006761-2,016,011), and written informed consents were obtained from the patients.

### Surgical technique

All patients were operated by the same surgeon (JD). Under general or spinal lumbar anesthesia, all patients underwent arthroscopic exploration and necessary management of intraarticular lesions before open ligament repair. Suture repair was performed for patients with mid- substance ligament ruptures, and suture repair with anchor was performed for patients with ruptures near the ligament attachment. OCLs were carefully measured with a marked probe and classified according to Ferkel and Cheng’s classification [16]. Debridement bone marrow stimulation was used for stage B-C OCLs treatment, and bone marrow stimulation for stage D-F OCLs. Other lesions (synovial hyperplasia, impingement, loose bodies) were also treated under arthroscopy. The ligament tear site was then assessed to guide the incision location.

**Fig. 1:**
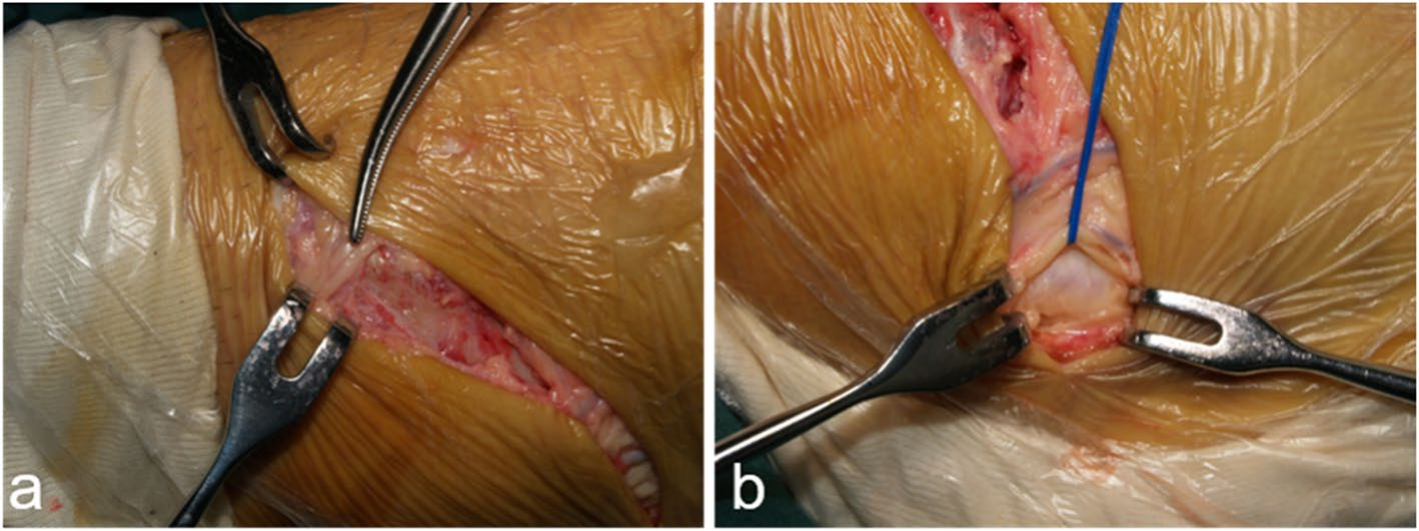
Care was taken to avoid the intermediate branch of the superficial peroneal nerve (**a**) and sural nerve (**b**)

**Fig. 2:**
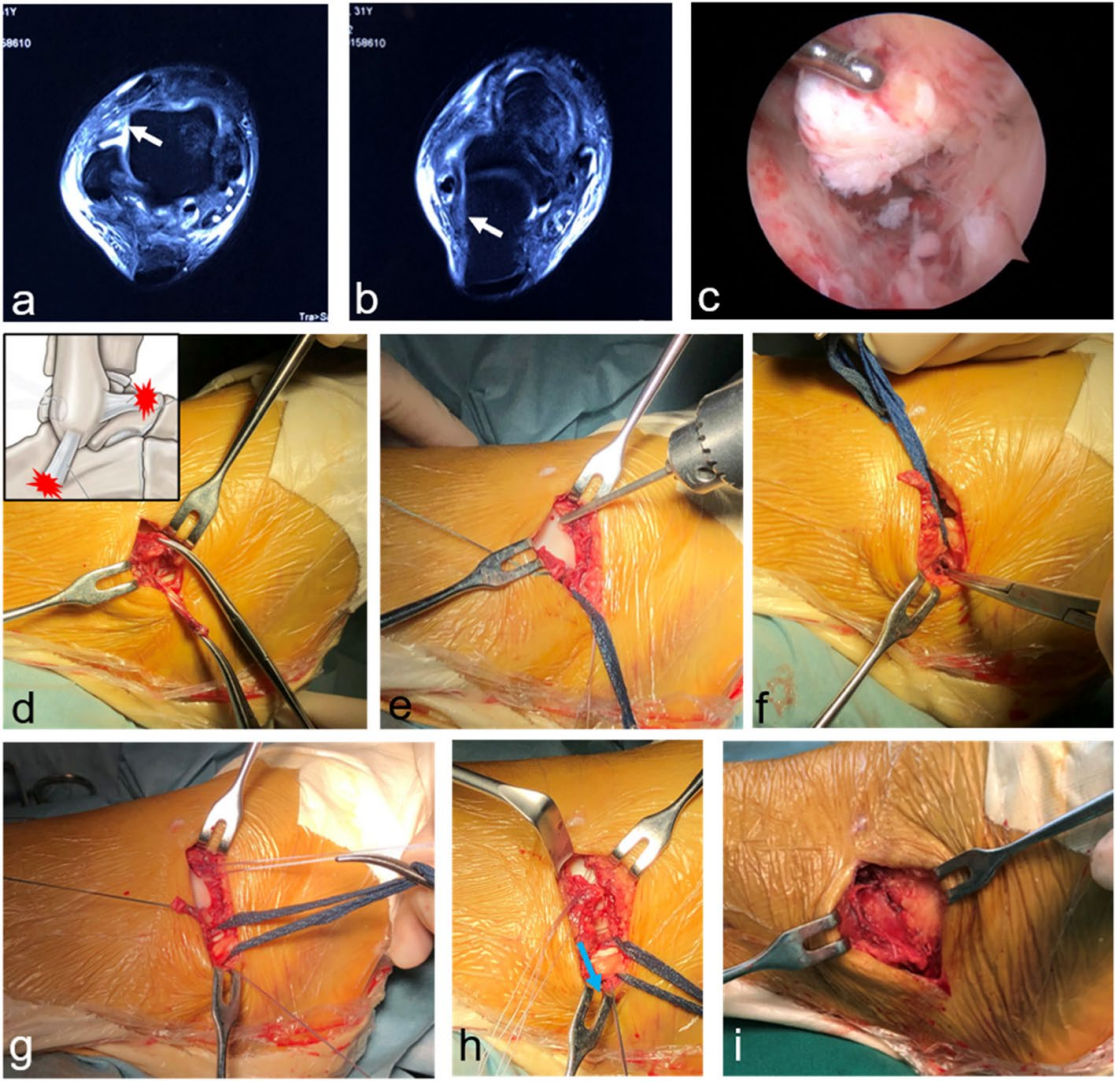
A patient with an anterior talofibular ligament (ATFL) tear at the talar site (**a**) and a calcaneofibular ligament (CFL) tear at the calcaneal site (**b**), which were identified in magnetic resonance images (MRI). The end of the ruptured ATFL was identified under arthroscopy (**c**). The ruptured ends were isolated (**d**), and the suture anchor was inserted into the insertion site (**e**,**f**). The ligament was then braided and pulled underneath the peroneal tendon (blue arrow) and fixed with the anchor wire (**g**, **h**). The extensor retinaculum was then sutured to the fibular periosteum (**i**)

**Fig. 3:**
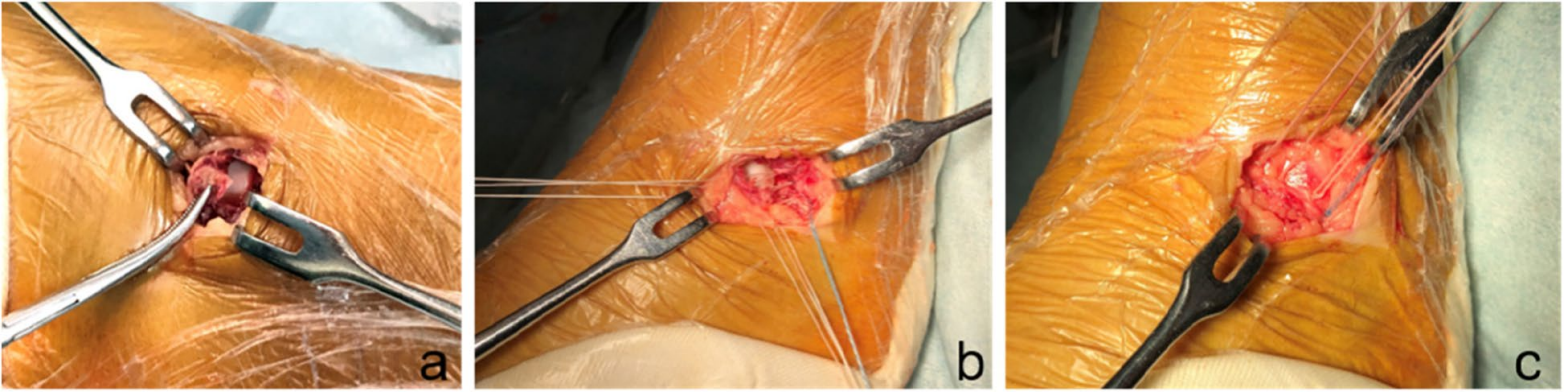
A patient with avulsion fractures with a diameter of < 1 cm. We carefully peeled the ligaments from the surface and resected the fracture fragment then sutured the ligament tissue to the bone surface with anchors

After ankle arthroscopy, a slightly curved longitudinal incision was made 3–4 cm above the distal tip of the fibula, which was guided by the location of ligament tear observed under arthroscopy. The incision for talar tears of the ATFL was more prone to anterior, and the incision for calcaneal tears of the CFL was more prone to distal. Care was taken to avoid the intermediate branch of the superficial peroneal nerve and sural nerve (Fig. 1). The ATFL and CFL were exposed to evaluate the injured site. Mid- substance ruptures were sutured using a 2–0 polyester braided wire. Proximal ruptures were repaired using 1.8-mm diameter suture anchors (Mitek Mini–GII, Johnson & Johnson, NJ), which were positioned at the anatomic insertion site in the distal fibula, anterolateral talus, or calcaneus. For distal ruptures, a suture anchor was inserted at the calcaneal insertion site. Then the ligament was braided and pulled underneath the peroneal tendon and fixed with the anchor wire (Fig. 2). For avulsion fractures with a diameter of < 1 cm, we carefully peeled the ligaments from the surface and resected the fracture fragment, then sutured the ligament tissue to the bone surface with anchors (Fig. 3). The extensor retinaculum was then sutured to the fibular periosteum. ROM, anterior drawer, and talus tilt were assessed, and finally, the ankle joint was immobilized with a splint in slight dorsiflexion and eversion.

Patients were classified into three groups according to the rupture site, including proximal group (ruptures at the fibular end of two ligaments), mid-substance group (ruptures of the mid-substance of two ligaments) and distal group (rupture near the talar or calcaneal insertion site). Ruptures involving the fibular side and the mid-substance were classified as the proximal group; ruptures at the talar/calcaneal side and mid-substance were classified as the distal group; and ruptures involving the talus/calcaneus and the fibular side were grouped according to the most severely injured site.

### Postoperative rehabilitation

A splint was applied for the first 3 weeks postoperatively, without weight-bearing. The splint was then replaced with an ankle brace, which remained in place until 8 weeks postoperatively. Passive flexion–extension ROM exercises were started from week 3, and varus traction exercise was allowed from week 5. Full weight-bearing was allowed between weeks 3–5, and patients were advised to return to sports 2–3 months postoperatively. For patients with OCLs, the splint or brace was removed twice a day for full-range continuous passive motion (CPM) exercise from weeks 2–6. For these patients, partial weight-bearing was allowed 6–8 weeks postoperatively, with full weight-bearing starting from 8–12 weeks postoperatively.

### Clinical outcomes evaluation

The present study was a retrospective investigation. All eligible patients were contacted in October 2021 (3–11 years after operation) for final follow up. Subjective outcomes were evaluated before surgery and at final follow-up, including the pain visual analog scale (VAS) [17], American Orthopaedic Foot & Ankle Society (AOFAS) score [18], Tegner activity score [19, 20]. Time to return to sports and resumption of pre-injury sports level were recalled at final follow-up. Participation at 85% or higher of the pre-injury level was recorded as resuming pre-injury sports level. Postoperative complications were collected based on medical records at routine clinic check and recalls at final follow-up.

### Statistical analysis

Data were analyzed using SPSS Statistics version 23.0 (IBM Corp., Armonk, NY). The chi-square test or Fisher’s exact probability test was used for categorical outcomes, while the paired samples t-test and nonparametric test were used to determine the subjective scores of preoperative and follow-up endpoints and the site of ligament injury on postoperative outcomes. Differences were considered significant at *p* < 0.05. PASS 11.0 (NCSS, US) was used to calculate the sample size. Post-hoc power analysis revealed that a sample size of 115 could achieve 90% power to detect the observed group difference in return-to-sport.

## Results

Of the 130 patients who met the study inclusion criteria, 117 patients (90%) were available for the final follow-up. All eligible patients were included. Post-hoc power analysis revealed that a sample size of 115 could achieve 90% power to detect the observed group difference in recovery of sports. Suture repair was performed for 41 (35%) patients with mid-substance ligament ruptures, and suture repair with anchor was performed for 76 (65%) patients with ruptures near the ligament attachment. Among the 76 cases of injury near the ligament insertion, 48 cases were near the fibular end (including 6 cases of ruptures involving the fibular side and the mid-substance) and 28 cases were near the talus or calcaneus insertion site (including 10 cases of ruptures at the talar or calcaneal side and mid-substance). 12 (10%) patients were professional athletes, namely 5 football players, 4 judo players, 1 tennis player, 1 volleyball player, and 1 hockey player. The other 105 patients included college team athletes, semi-professional athletes and coaches. Under arthroscopy, the intra-articular lesions were treated for 28 (24%) patients with OCLs, 12 (10%) with avulsion fractures, and 10 (8%) with osteophytes (Table 1).

**Table 1:** Demographic characteristics of the 117 patients

Characteristics	Patient cohort
Sex, *n* (%)	
Male	85 (73)
Female	32 (27)
Age, years	26.5 ± 10.1
BMI, kg/m^2^	24.1 ± 3.3
Injury time, days	7.7 ± 4.2
Follow-up, months	46.4 ± 16.1
OCLs, *n* (%)	
B-C	15 (13)
D-F	13 (11)
Avulsion fracture, *n* (%)	
Fibular	10 (8)
Talus	1(1)
Calcaneus	1 (1)
Combined medial ligament injury, *n* (%)	3 (3)
Osteophyte, *n* (%)	10 (8)
Location of lateral ligament rupture, *n* (%)	
Proximal group	48(41)
Middle group	41 (35)
Distal group	28 (24)
Fixation technique, *n* (%)	
Suture anchor	76 (65)
Simple suture	41 (35)

*BMI* Body Mass Index, *OCLs* Osteochondral Lesions

A total of 130 patients met the study inclusion criteria, of whom 117 patients (90%) were available for the final follow-up. 12 (10%) patients were professional athletes (5 football, 4 judo, 1 tennis, 1 volleyball, and 1 hockey player). The other 105 patients included college teams, coaches, and amateur athletes. Suture repair was performed for 41 (35%) patients with mid-substance ruptures, and suture repair with anchor was performed for 48 (41%) proximal and 28 (24%) distal ruptures. Under arthroscopy, the intra-articular lesions were treated for 28 (24%) patients with OCLs, 12 (10%) with avulsion fractures, and 10 (8%) with osteophytes (Table 1)**.**

At a mean follow-up of 46.4 ± 16.1 months after surgery, all subjective scores were significantly improved from the pre-operative level (*p* < 0.001), with a postoperative VAS, AOFAS and Tegner scores of 0.35 ± 0.90, 98.15 ± 3.72 and 5.03 ± 0.78, respectively ( Table 2). A total of 14 (12%) patients reported mild ROM restriction (< 10°), of whom 9(64%) were plantar flexion limitation. 7 (6%) patients experienced transient numbness on the lateral side of the foot related to superficial peroneal nerve irritation, which resolved in 5 patients after 6 months.

**Table 2:** Subjective outcomes before surgery and at final follow-up

	pre-operation	post-operation	*P* value
VAS pain	6.13 ± 1.37	0.35 ± 0.90	< 0.001*
AOFAS	24.52 ± 8.04	98.15 ± 3.72	< 0.001*
Tegner	0.83 ± 0.61	5.03 ± 0.78	< 0.001*

*VAS* Visual Analog Scale, *AOFAS* American Orthopaedic Foot & Ankle Society

^*^Statistically significant difference (*p* < 0.05)

The average time to return to sports was 4.34 ± 1.11 months after surgery.90 ± 13% resumed pre-injury sports level. 11 (92%) of the 12 professional athletes fully resumed their pre-injury competitive levels and participated in national and international competitions. The distal group reported the smallest proportion (86% ± 13%) of resumption of pre-injury sports level, compared with 93% ± 12% for the proximal group and 89% ± 14% for the middle group (*p* = 0.037). Time to return to sports was significantly longer for the distal group, with an average of 4.59 ± 1.27 months compared to 3.99 ± 1.09 months for the proximal group and 4.58 ± 0.90 months for the middle group (*p* = 0.009). The distal group returned to sport about 3 weeks later than the middle and proximal groups. The proximal group showed the highest rate of ROM restriction and the distal group showed the highest rate of sprain recurrence but with no significant difference (*p* > 0.05). There was no significant difference in VAS, AOFAS, or Tegner scores between the three groups ( Table 3).

**Table 3:** Effect of the location of ligament tear on postoperative outcomes

	Proximal group	Middle group	Distal group	*P* Value
	(*n* = 48)	(*n* = 41)	(*n* = 28)	
Recovery of sports				
Percentage (%)	93 ± 12	89 ± 14	86 ± 13	0.037*
Time, months	3.99 ± 1.09	4.58 ± 0.90	4.59 ± 1.27	0.009*
ROM restriction, *n* (%)	7 (50)	4 (31)	3 (19)	0.117
Sprain recurrence, *n* (%)	4 (40)	4 (40)	2 (20)	0.809
Satisfaction (%)	83 ± 8	84 ± 9	83 ± 12	0.901
VAS pain				
pre-operation	6.15 ± 1.27	6.12 ± 1.36	6.11 ± 1.59	0.922
post-operation	0.23 ± 0.78	0.27 ± 0.71	0.68 ± 1.25	0.13
AOFAS				
pre-operation	25.25 ± 8.35	24.37 ± 7.87	23.50 ± 7.92	0.669
post-operation	98.92 ± 2.74	97.56 ± 4.50	97.68 ± 3.82	0.373
Tegner				
pre-injury	5.35 ± 0.81	5.37 ± 0.70	5.44 ± 0.77	0.867
post-operation	5.02 ± 0.76	5.15 ± 0.65	4.86 ± 0.97	0.209

*ROM* Range Of Motion, *VAS* Visual Analog Scale, *AOFAS* American Orthopaedic Foot & Ankle Society

^*^Statistically significant difference (*p* < 0.05)

## Discussion

The most important finding of this study was that ankle arthroscopic treatment followed by anatomic ligament repair achieved good mid-term results and could be a reliable procedure for patients requiring return to high demanding sports after severe acute ankle sprains. Distal ruptures near the talar or calcaneal end were associated with delayed return to sports and inferior performance at pre-injury sports level. Patients with distal ruptures returned to sports about 3 weeks later than other patients.

The results showed favorable mid-term outcomes of the procedure, restoring good ankle stability and resuming pre-injury sports for most patients. The results were parallel to previous studies of ligament repair for acute ankle sprains [21]. Although conservative treatment is often performed for grade I & II acute ankle ligament injury [22], the benefit of surgery is gaining evidence for grade III lateral ligament injuries and patients with requirements to return to highly intensive sports. White et al. [23] followed up 42 players undergoing acute lateral ankle ligament repair and the results showed that lateral ligament reconstruction with the modified Broström method was a safe and effective treatment for acute severe ruptures, providing a stable ankle and expected return to sports at approximately 10 weeks. Surgery was inclined to be used for combined ATFL and CFL ruptures [23]. Samoto et al. [24] assessed the results of nonoperative treatment of acute lateral ligament injury according to its severity, and the result was unsatisfactory in those with combined injuries of the ATFL and CFL. All patients enrolled for this study had both ATFL and CFL ruptures requiring return to high demanding sports, which could be a good indication for this procedure.

Excellent outcomes may also be attributed to the use of arthroscopy, which facilitate thorough exploration and proper management of intra-articular lesions, especially for OCLs [8, 9]. In the present study, OCLs were found in 28 (24%) patients, of which there were 13 free osteochondral fragments. The OCLs have been widely recognized as a negative predictor on clinical outcomes of the lateral ankle ligament repair [25–27].

This study also suggested that the location of ligament rupture could affect return-to-sport. Patients with distal ruptures were relatively less likely to resume pre-injury sports level. They also required about 3 weeks longer to return to sports. One of the reasons of the results might be attributed to more involved CFL ruptures in the distal group. Our results showed that proximal injuries were mainly in the ATFL, while distal injuries were mainly in the CFL. Notably, some studies indicated that the recovery after CFL injury was worse than that of ATFL injury [27]. Regarding anatomy, the ATFL that is a flat quadrilateral ligament and incorporated in the joint capsule while the CFL is a cylindrical ligament, and difficulties in recovery relate to its anatomical location beneath the peroneal tendons [28]. Therefore, the anatomical position of ATFL is shallower and flatter, which may be easier to repair than CFL [11]. Regarding blood supply, the lateral talar body has worse vascularization than other parts of the talar body, and the lateral blood vessel density is lower than that for other parts, which may explain why outcomes after ruptures to the end of the talus is not as good [29]. However, there is no direct evidence to prove that the blood supply varies at different location of the ligaments and further research is needed.

In the present study, the middle group showed a similar late postoperative return to sport time and proportions as the distal group. The main reason might be due to that both of the ATFL and CFL in the middle group were ruptured at the substance of ligament with greater tissue instability. In addition, the middle group was fixed with simple sutures rather than anchors, which might weaken the strength of the ligaments in the early postoperative period, thereby slowing down the speed of recovery.

It was also founded that 10% (12/117) of the patients had avulsion fractures, and the results showed that removing avulsion fragments with a diameter of < 1 cm did not affect ligament stability. Lateral ankle avulsion fracture was reported to have a potential negative impact on postoperative rehabilitation [30], and patients with avulsion fracture should be informed of the risk of recurrent sprain and subsequent ankle instability; careful follow-up is needed for these patients [31]. Fixing or removing the avulsed fragment is determined by the size of fragment [32]; fragments can be removed for small avulsion fractures [33].

Although the anatomic ligament repair surgery in this study resulted in overall fast postoperative recovery, the average return to sports time of 4 months was relatively long, especially for athletes. Recently, prosthetic augmentation techniques using suture tape augmentation became an introduced procedure, which could expedite the recovery process as well as strengthen the repair construct and protect it from future injury [34, 35]. The internal brace and other biologics that promote ligament repair might be applicable for operative treatment in acute ligament injury to further accelerate the time of rehabilitation and return to sports.

Therefore, the present research reported the mid-term follow-up study of the concurrent arthroscopy and open lateral ankle ligament repair for the acute ankle sprain and analyze the impact of the location of ligament rupture on the outcomes. The relatively large sample size could provide a reliable conclusion on the effectiveness of the operation and the potential problems. The results of the impact of the rupture site provided a reference for an optimal and personalized postoperative rehabilitation. Patients with proximal injuries might be encouraged to perform more aggressive rehabilitation. Those with ligament rupture near the calcaneal or talus site could return to sport at about 14 weeks while the rupture near the fibular site at about 12 weeks. It should be also noted that the surgery prefers to be used in those with severe ligament injury and high sports demanding in spite of the excellent postoperative outcomes. Most ankle sprains were recommended conservative treatment and rehabilitation training, and satisfactory results could be obtained.

There are still some limitations about this research. First, it was retrospective rather than prospective research with no comparative groups undergoing conservative treatment or isolated ligament repair without arthroscopy. In addition, patients were divided into three groups according to the rupture sites, which was not absolutely strict, because the tear of the ligament was usually reported as a cauda equina rather than a simple avulsion from the insertion site. Therefore, the groups in the present study were determined according to the location of the most severe tear, which basically reflected the area of the main injury.

## Conclusions

Ankle arthroscopy followed by open anatomic ligament repair is a reliable procedure for patients requiring return to high demanding sports after severe acute ankle sprain. Rupture in the substance and near the talar or calcaneal side appeared to weaken the sports resumption and delay about 3 weeks of sports recovery.

## Data Availability

All data generated or analyzed during this study are included in this article.
